# *Fusarium* Mycotoxins Are Frequently Detected in Oat Grains and Oat Foods and T-2, HT-2 and HT-2-Glucoside Are Highly Bioaccessible in Oat Porridge

**DOI:** 10.3390/toxins18070295

**Published:** 2026-07-07

**Authors:** Margaret-Jane Gordon, Noshin Daud, Louise Cantlay, Silvia W. Gratz

**Affiliations:** Rowett Institute, University of Aberdeen, Foresterhill Health Campus, Aberdeen AB25 2ZD, UK; m.j.gordon@abdn.ac.uk (M.-J.G.); noshin.daud@abdn.ac.uk (N.D.); l.cantlay@abdn.ac.uk (L.C.)

**Keywords:** *Fusarium*, trichothecenes, masked mycotoxins, prevalence, dietary exposure

## Abstract

The presence of *Fusarium* mycotoxins is an intractable problem in cereal production, with T-2 and HT-2 posing a particular issue in oats. This study assesses the prevalence of *Fusarium* mycotoxins in unprocessed and de-hulled oats and oat food products and quantifies the bioaccessibility of T-2, HT-2 and HT-2-glucoside from oat porridge in vitro. Twenty unprocessed food oat samples were de-hulled and mycotoxins quantified in unprocessed grains, hulls and groats using LC-MS/MS. Seventy-seven oat food products (40 porridge samples, 10 granola and muesli samples, 11 oat biscuits, 11 oatcakes, 5 infant cereals) were analysed for mycotoxins. Two oat porridge samples were tested for bioaccessibility using Infogest 2.0 method followed by faecal microbiota incubations. T-2/HT-2 and their glucosides were the most prevalent mycotoxins in unprocessed food oats (85–100%). DON was also highly prevalent (70%) while DON-glucoside, NIV, ZEN and their glucoside were less frequently detected. Reduction of 89–100% was achieved by de-hulling oats for most mycotoxins except DON-glucoside (77% reduction). Oat food samples were also frequently contaminated with T-2+HT-2 (prevalence porridge 90%, granola and muesli 40%, oat biscuits 36%, oatcakes 73%) while no mycotoxins were detectable in infant cereal foods. Upon in vitro digestion, 73–82% of free and glucosylated T-2/HT-2 were readily bioaccessible, underlining their importance for human dietary exposure. The study highlights that T-2/HT-2 contamination is prevalent in oats and carry-over into food products cannot be completely avoided, potentially leading to mycotoxin exposure in oat food consumers.

## 1. Introduction

Mycotoxin contamination of cereal grains is an intractable and global issue and poses a serious concern for food safety [1]. Mycotoxins are toxic secondary metabolites produced by a wide range of fungi infecting cereal crops, and global patterns of fungal infection are impacted by climate change and severe weather events [2,3]. Amongst the field fungi, *Fusarium* species pose a major problem in small grain cereals, causing a cereal disease called Fusarium Head Blight and some fungi of this species are known to produce a range of mycotoxins [3]. The main groups of *Fusarium* mycotoxins detected in small grain cereals such as wheat, barley and oats comprise trichothecenes, zearalenone (ZEN) and fumonisins [4,5]. The most commonly detectable type A trichothecenes include T-2 and HT-2 toxins (T-2, HT-2) as well as the less prevalent mycotoxins diacetoxyscirpenol and neosolaniol (DAS, NEO), while type B trichothecenes include deoxynivalenol and nivalenol (DON, NIV) [5]. In addition to these fungal parent mycotoxins, cereal grains are commonly co-contaminated with plant-derived modified mycotoxin metabolites including glucose conjugates of T-2, HT-2, DAS, DON, NIV and ZEN (T-2-glucoside, T-2-Glc; HT-2-glucoside, HT-2-Glc; diacetoxyscirpenol-glucoside, DAS-Glc; deoxynivalenol-glucoside, DON-Glc; nivalenol-glucoside, NIV-Glc; zearalenone-glucoside, ZEN-Glc) [6]. Type A trichothecenes pose a particular problem in oats with over 90% of grain samples found to be contaminated with T-2 and HT-2 in several studies [7,8,9,10,11,12,13,14,15]. Co-contamination with type B trichothecenes and other mycotoxins is also reported at lower prevalence. Trichothecenes are intestinal toxins and immunotoxins with type A trichothecenes being more potent toxins than type B compounds [5]. ZEN is an oestrogenic compound known to reduce fertility in farm animals [4]. Due to the known toxicity of these mycotoxins, regulatory bodies around the world have introduced maximum permitted levels for mycotoxins in raw commodities and foods to minimise mycotoxin exposure through contaminated food. The European Commission has recently updated its regulations for mycotoxins in foods in 2023/2024 and for the first time introduced maximum levels (ML) for T-2 and HT-2 toxins in cereals and foods (Table 1), replacing previous indicative levels [16,17,18].

The introduction of regulatory limits for T-2/HT-2 in cereal foods has generated interest in the prevalence and levels of these mycotoxins in oats and oat food products, with the European Food Safety Authority (EFSA) [17] as well as the UK Food Standards Agency (FSA) publishing specific calls for evidence of these mycotoxins in unprocessed food oats and oat foods. Furthermore, knowledge of the fate of free and modified mycotoxins upon ingestion is limited [19]. To predict bioaccessibility, which is the portion of a food contaminant released from food matrix during gastric and small intestinal digestion and theoretically available for uptake into systemic circulation, in vitro models have been used [20]. High bioaccessibility of mycotoxins aflatoxin B_1_ (AFB_1_), ochratoxin A (OTA), DON and T-2/HT-2 have been reported from naturally contaminated and artificially spiked food matrices including bread and biscuits [21,22]. However, information on the presence of T-2 and HT-2 in foods with high oat content is limited and no study has to date investigated their bioaccessibility. This study aims to address this knowledge gap by assessing the prevalence of mycotoxins in unprocessed food oats, de-hulled oats and oat food products and by predicting their bioaccessibility from oat porridge in vitro. The study monitors free and glucosylated mycotoxins and discusses the findings in relation to exposure risk assessments.

## 2. Results and Discussion

Oats are an important cereal commodity and are widely promoted as healthy foods, especially for breakfast cereals. Moderate consumption of porridge and muesli was correlated with a reduced risk of all-cause mortality [23] in a recent large-scale prospective study using UK Biobank data of 186,168 participants who recorded their food intake in 24 h recall during 2006–2010. The study also reports that the most commonly consumed breakfast cereals amongst adults are porridge (23.9% of participants) and muesli (22.1% of participants), highlighting the importance of oat products as part of a healthy diet in UK adults. It is therefore paramount that potential health risks related to mycotoxin contamination in oats and oat foods are well understood and managed.

### 2.1. Mycotoxin Contamination of Unprocessed Oats and Oat Fractions

Unprocessed oat grains consist of oat kernels (or groats) and firmly attached, inedible husks (or hulls). After de-hulling, oat groats contain whole oat kernels with outer layers and germs remaining [24]. De-hulling of unprocessed oats (100 g) yielded 31.3 ± 4.3 g hulls, 59.7 ± 4.3 g groats (de-hulled kernels) and 9.1 ± 2.1% waste with incomplete separation of hulls and groats. Results are in line with literature [24], reporting 23–31% of hull in laboratory de-hulling of oats.

Major *Fusarium* mycotoxins were highly prevalent in unprocessed whole oats and hulls, but less frequently detectable in de-hulled groats (Table 2). Free and glucosylated T-2 and HT-2 were most frequently detected in whole oats (85–100% prevalence) with 10% of samples exceeding the recent maximum levels for T-2+HT-2 permitted in unprocessed oats (Table 1). Free and glucosylated T-2 and HT-2 were also the most prevalent mycotoxins in oat hulls (80–100%) and groats (25–55%). Type B trichothecenes and ZEN were less frequently detected. DAS and DAS-Glc were monitored but not quantified. DAS was detectable in one whole oat and two hull samples, DAS-Glc was detectable in two hull samples, not co-occurring with DAS. During laboratory de-hulling, Meyer et al. [23] also found significant reduction of T-2+HT-2 in oat groats compared to unprocessed oat grains. The study also detected fungal DNA of *Fusarium langsethiae,* a major T-2/HT-2 producer, in unprocessed oats and oat groats and found levels of fungal DNA positively associated with levels of T-2+HT-2 in both fractions. This demonstrates that *F. langsethiae* infection is most prevalent in hulls, but the fungus also penetrates into oat groats and produces mycotoxins [24].

Reduction factors were calculated as the ratio of mycotoxin concentrations in groats or hulls to the concentration in whole oats, back-calculated from concentrations in fractions (hulls + groats) and the masses of each oat fraction to avoid variation due to the heterogeneity of mycotoxins in whole oats [25]. Over 90% of total mycotoxins were removed with the hull for all detectable free and glucosylated mycotoxins with the exception of DON-Glc where only 77.1% were removed (Table 2). The mean reduction factors for DON (93%) as well as T-2 and HT-2 (93 and 99%) are broadly in line with reports in the literature. Edwards reported reduction factors of 58–98% (mean 89%) for the sum of T-2+HT-2 in 66 oat samples [26] while Tittlemier et al. [25] found HT-2 in 21% of hulls of 14 oat samples, but not in any groats, indicating that 100% of HT-2 were associated with hulls. Similarly, Meyer et al. [24] reported a mean reduction factor of 84% for T-2+HT-2 in 32 oat samples while Dropa et al. [27] reported a reduction of T-2 and HT-2 of 48 and 58% during commercial de-hulling of oats. Mean reduction factors reported for DON in oats range from 72% to 87% [24,25], but only 48% of DON-3G were associated with hulls, again in line with findings presented here.

### 2.2. Mycotoxin Contamination and Co-Contamination in Porridge Oats

Porridge oat samples (*n* = 40, purchased from retailers in 2024 and 2025 to increase sample size) were analysed for *Fusarium* mycotoxins. The prevalence of type A trichothecenes was high in porridge oat samples with 90% of all samples >LOQ for T-2 at mean concentrations of 9.4 ng/g porridge oats as purchased (Table 3).

The prevalence of HT-2 and HT-2-Glc were lower (67.5 and 35.0%, respectively) but observed concentrations were higher than T-2. Mean mycotoxin levels in retail samples from 2024 and 2025 were not significantly different except for HT-2-Glc (mean 2.7 ng/g in 2024 and 17.4 ng/g in 2025, *p* = 0.011). It is unclear why this plant metabolite of HT-2 was higher in samples from 2025 as no such differences were observed for fungal parent mycotoxins HT-2 or T-2 in this study. No reference standards are currently available for the quantification of HT-2-Glc and future work is needed to better understand the prevalence of this modified mycotoxin and the factors affecting its production in oats and carry-over into oat food products. T-2-Glc, DAS and DAS-Glc were not quantifiable (<LOQ) in any sample. Type B trichothecenes DON and NIV were quantifiable in 10.0–12.5% of samples with maximum concentrations of 283.9 and 36.8 ng/g detected for DON and NIV. DON-Glc was not quantified but detectable in five samples, co-occurring with DON while NIV-Glc was not detectable in any sample. ZEN was not quantifiable in any sample and ZEN-Glc in one sample. Results are broadly in line with the literature reports on oat porridge, oat flakes and oatmeal. A UK survey of oat cereals and oat drinks found DON as the most prevalent mycotoxin in porridge oats (*n* = 6, prevalence 100%, range 27.2–355 ng/g) followed by DON-Glc (prevalence 83%, range 20.7–190 ng/g), HT-2 (prevalence 67%, range 16.4–62.8 ng/g) and T-2 (prevalence 17%, 10.4 ng/g) while T-2-Glc was <LOQ in all samples and HT-2-Glc was not analysed [28]. A large study assessing European oat products [29] also reports T-2+HT-2 frequently occurring in oat products including oat flakes (*n* = 259, prevalence 73%, mean 17 ng/g) and oatmeal (*n* = 105, prevalence 34%, mean 11 ng/g) with T-2 at approximately 52% of HT-2. EFSA has published two scientific opinions on T-2/HT-2 toxins. In the 2011 assessment [30], levels are reported in oat flakes (T-2+HT-2 *n* = 1089, prevalence 47%, LB mean 14 ng/g; T-2 *n* = 1003, prevalence 42%, LB mean 3.5 ng/g; HT-2 *n* = 1003, prevalence 69%, LB mean 10 ng/g). A subsequent detailed analysis [31] of occurrence data on T-2 and HT-2 submitted to EFSA (2011–2016, 20 European countries) comprised grains and grain-based products (*n* = 7126 data points for T-2 and *n* = 5711 data points for HT-2). The highest mean concentrations were measured in oat grains for human consumption (LB mean = 40.2 ng/g for T-2, LB mean = 91.4 ng/g for HT-2). Oat milling products showed the highest mean HT-2 concentrations (LB mean = 16.6 ng/g) and a high prevalence of HT-2 (61%). While prevalence and levels of T-2 and HT-2 vary between reports, the overall picture of frequent contamination of oats and oat products with T-2 and HT-2 and higher levels of HT-2 compared to T-2 are consistent. T-2 Glc is rarely detected while HT-2-Glc has been confirmed as highly prevalent in oats [6].

**Figure 1:**
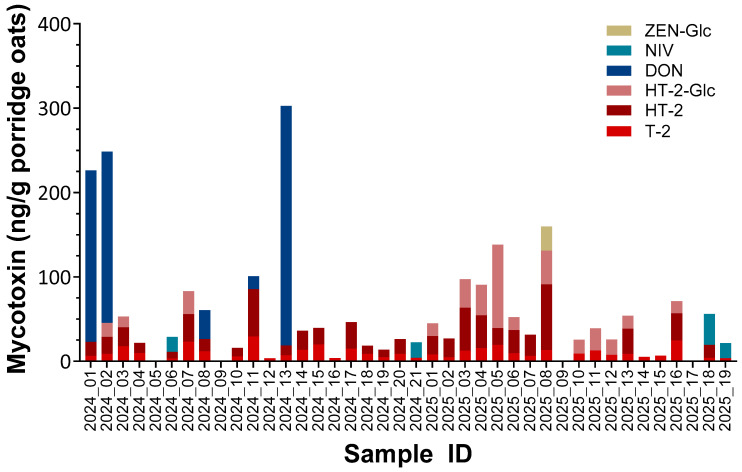
Co-contamination of individual porridge oat samples (2024 and 2025) with multiple *Fusarium* mycotoxins. Values presented as ng/g porridge oats as purchased.

Co-contamination of oats with multiple mycotoxins was frequently observed with 33/40 samples contaminated with two or more mycotoxins while only four samples were contaminated with one mycotoxin (T-2) and three samples contained no detectable mycotoxins (Figure 1). Co-contamination with two or three type A trichothecenes was most commonly observed (25/40) while 4/40 samples contained two or three type A and type B mycotoxins. Results are in line with previous reports of frequent co-occurrence of T-2 and HT-2, often in combination with DON or NIV [9,28]. EU maximum levels (ML) for DON and T-2+HT-2 (Table 1) were not exceeded in any oat porridge sample.

### 2.3. Mycotoxin Contamination in Oat Foods for Adults and Infants

Additional oat food categories, which are important in UK oat consumption, have been included in this survey. T-2 and HT-2 toxins were the most prevalent mycotoxins in granola and muesli samples (*n* = 10, prevalence 40 and 40% for T-2 and HT-2), oat biscuits (*n* = 11, prevalence 27 and 36%), and oatcakes (*n* = 11, prevalence 46 and 64%). HT-2-Glc was also prevalent (40, 36 and 27% in oat biscuits, granola and muesli, and oatcakes, respectively, Table 4). No mycotoxins were detectable in any of the infant cereal foods included in this survey. Results for granola and muesli samples are in line with published reports from EFSA [30] in muesli (T-2 *n* = 104, prevalence 73%, LB mean 1.7 ng/g; HT-2 *n* = 108, prevalence 57%, LB mean 6.0 ng/g; T-2+HT-2 *n* = 249, prevalence 88%, LB mean = 5.6 ng/g) and in breakfast cereals (T-2 prevalence 27%, LB mean = 2.07 ng/g; HT-2 prevalence 40%, LB mean = 5.34 ng/g) [30]. In contrast, FSS [28] reported no T-2, HT-2 or T-2-Glc in granola and muesli samples (*n* = 3), oat biscuits (*n* = 3) or oatcakes (*n* = 3). Levels of T-2+HT-2 found in this study could potentially be above EU maximum levels (see Table 1) in 1/11 biscuits and 2/11 oatcake samples, but none of the granola and muesli samples. The previous smaller FSS survey of UK retail samples reported 0/15 oat food products exceeding indicative ML for T-2+HT-2 [28]. The modified mycotoxin T-2-Glc was not quantifiable in any samples, confirming the earlier FSS report [28]. HT-2-Glc was found in 10/32 samples, always co-occurring with HT-2 at ratios of 64–299% of HT-2. No commercial reference standard is available for HT-2-Glc, but results from this survey clearly indicate significant co-occurrence of this modified mycotoxin in oat foods.

Results for infant cereal foods are in line with a recent study analysing 39 breakfast cereals and 25 cereal bars marketed for infants and young children for 20 mycotoxins from Portugal, which did not find DON, T-2 or HT-2 in any sample analysed [32]. However, the authors did not specify the composition of the cereals in the study and their oat content. In contrast, Al-Taher et al. [33] analysed oat-based infant cereals from the US and found DON in 15/20 samples (2.5–146.5 ng/g), T-2 in 8/20 (0.6–2.3) and HT-2 in 4/20 (2.4–9.6 ng/g). In Europe, EFSA [30] report frequent contamination of cereal-based infant foods (T-2 *n* = 141, prevalence 55%, LB mean 0.88 ng/g; HT-2 *n* = 140, prevalence 36%, LB mean 2.7 ng/g; T-2+HT-2 *n* = 390, prevalence 71% LB mean 2.7 ng/g) and FSA [34] report very high levels of T-2 in infant cereals (*n* = 2, mean 110.6 ng/g). The conflicting results of mycotoxin levels in infant cereal foods reported in the literature need further investigation to improve our understanding of contamination in this important food group.

### 2.4. Bioaccessibility of T-2, HT-2 and HT-2-Glc from Oat Porridge

Bioaccessibility is presented as the proportion of each mycotoxin that is available to be released through the gastrointestinal tract from the food matrix and then potentially absorbable. Two naturally contaminated porridge oat samples were used to determine the bioaccessibility of T-2, HT-2 and HT-2-Glc. Both samples were boiled as oat porridge prior to analysis and in vitro digestion. Both oat porridge samples contained higher levels of HT-2 compared to T-2 and HT-2-Glc at ratios of 83 and 50% of free HT-2 (Table 5). T-2-Glc and other mycotoxins measured (DON, NIV, DAS, ZEN and their glucosides) were <LOQ in both porridge oat samples.

Upon simulated upper GI digestion, the majority of free T-2 and HT-2 were released from both oat porridge samples (bioaccessibility 81.9 and 75.4% for T-2 and 64.5 and 57.6% for HT-2 in oat porridge 1 and 2, respectively). Simulation of the lower GI release of mycotoxins from digested oat porridge by human faecal microbiota resulted in very minor increases in bioaccessibility (3.4 and 2.7% for T-2; 6.6 and 7.2% from HT-2 in oat porridge 1 and 2, respectively). High bioaccessibility of T-2 and HT-2 has also been reported in other studies assessing baked foods. De Angelis et al. [22] reported lower bioaccessibility of T-2 (42 and 34%) compared to HT-2 toxin (91 and 98%) from spiked wheat bread (T-2 104 ng/g; HT-2 629 ng/g) and naturally contaminated wheat bread (T-2 47 ng/g; HT-2 at 893 ng/g). Lopez-Ruiz et al. [21] also reported lower bioaccessibility of T-2 (approx. 80%) than HT-2 (approx. 100%) from wheat biscuits spiked with 500 ng/g of each mycotoxin. Bioaccessibility strongly depends on the contaminant type, food source and matrix type [22] and these factors may contribute to the difference in our findings in oat porridge where higher bioaccessibility of T-2 was observed compared to HT-2.

The glucosylated mycotoxin HT-2-Glc was efficiently released from both oat porridge samples in the upper GI compartment (bioaccessibility of HT-2-Glc 96.2 and 89.3% for oat porridge 1 and 2, respectively). However, once HT-2-Glc was released from porridge, no further metabolism was observed, confirming that HT-2-Glc is stable and not hydrolysed by human digestive enzymes present in the upper GI tract [35]. Previous work also demonstrated that glucosylated trichothecene mycotoxins DON-Glc, NIV-Glc and T-2-Glc cannot be transported and absorbed through the human intestinal epithelium in vitro [36,37], but complete and rapid hydrolysis is observed in the presence of human gut microbiota [35,36,37,38,39,40]. Hence, EFSA recommends in their Scientific Option [41] a group-based TDI for the sum of T-2+HT-2+modified forms including their glucosides, assigning HT-2, T-2-Glc and HT-2-Glc a relative toxic potency factor of 1 compared to T-2. It can therefore be concluded that HT-2-Glc, which is efficiently released from oat porridge by small intestinal digestive enzymes, will be hydrolysed by colonic microbiota and free HT-2 becomes available for absorption, contributing to overall dietary exposure. Hence, total bioaccessibility of T-2 equivalents (sum of T-2+HT-2+HT-2-Glc, corrected for molecular weight) from naturally contaminated oat porridge in the entire GI tract (upper + lower GI) equates to 81.8% and 73.4% for the two oat porridge samples analysed.

### 2.5. Simplified Exposure Assessment

To illustrate the important contribution of free and glucosylated T-2/HT-2 to overall dietary mycotoxin exposure, we calculated the probable daily intake (PDI) of T-2 equivalents in different simplified exposure scenarios for children and adults. Mycotoxin occurrence in oat porridge was calculated as T-2 equivalents either as the sum of free T-2+HT-2 or as the sum of free T-2+HT-2+glucosylated HT-2-Glc, based on the mean mycotoxin levels across 40 porridge samples (Table 3). Hypothetical exposure to T-2 equivalents (probable daily intake, PDI) was estimated for children (assumed body weight of 18 kg, portion size of 20 g porridge/d) and adults (assumed body weight 70 kg, portion size 40 g/d). The PDI was compared to the tolerable daily intake (TDI) for the sum of T-2+HT-2+modified forms of 0.02 µg/kg body weight/d [41] to give a hazard quotient (HQ = PDI/TDI).

This simplified exposure assessment predicts the hypothetical exposure in children to exceed the safe level (TDI) by 1.5-fold if free T-2/HT-2 are considered (Table 6), which increases to 2-fold when glucosylated HT-2 is added to the exposure calculation. Glucosylated HT-2-Glc frequently co-occurs in unprocessed oats and oat foods (Table 2 and Table 3) and is highly bioaccessible from porridge, highlighting the importance of modified mycotoxin metabolites to contribute to overall dietary exposure in humans. For adults, the PDI is below the TDI in both exposure scenarios, indicating a negligible risk in adults.

These findings are in line with a growing body of evidence highlighting the increased risk in children to exceed the safe level of exposure to T-2/HT-2. A very comprehensive European risk assessment [31] estimated the chronic dietary exposure to the sum of T-2 and HT-2 in 35 dietary surveys from 19 different European countries. Overall, the chronic dietary exposure to the sum of T-2 and HT-2 was estimated to be 2- to 3-fold higher in the young population groups (infants, toddlers and other children) than that estimated for the adult population groups (adults, elderly and very elderly). Mean exposure estimates for T-2+HT-2 ranged from 8.5 (LB) to 62.1 (UB) ng/kg bw/d in children and from 2.5 (LB) to 26.4 (UB) in adults, comparable to the simplified exposure estimate presented here as PDI of 30.9 ng/kg bw/d for children and 16.0 ng/kg bw/d for adults (Table 6).

Similarly, a recent UK risk assessment for T-2/HT-2 [34] highlights a potential concern for consumer health, especially in infants and toddlers, and for some oat-based foods in adults and vegetarians/vegans (mainly oat porridge). This risk assessment uses UK food consumption data from the Diet and Nutrition Survey of Infants and Young children (2011) and the National Diet and Nutrition Survey (years 1–11) and occurrence data collected from the UK Food Standards Agency’s call for evidence. Data from the present study on levels of T-2 and HT-2 in porridge oats (Table 4, 2024 data), unprocessed oats [9] and oat fractions (Table 2) as well as unpublished data have been submitted to this call for evidence. Estimated chronic exposure to the sum of T-2 and HT-2, based on processed oat grains, found no risk associated with T-2/HT-2 exposure for any age group of the UK population. However, estimated chronic exposure based on ready-to-eat oat foods including porridge, muesli and infant cereals led to significant exceedances of TDIs, especially for infants and toddlers. These findings are in line with data from our current study as well as our previous urinary biomonitoring study which found a similar risk of high T-2/HT-2 exposure in UK children (mean HQ of 0.7–0.8 range 0.0–27.9) with T-2/HT-2 exposure linked to oat consumption [42]. A detailed French total diet study also reports mean exposure (lower bound) of children and adults to 14.5 and 8.9 ng/kg bw/d, which would result in a mean HQ of 0.73 for children and 0.44 for adults [43]. Taken together, findings point towards children being exposed to higher levels of T-2/HT-2 than adults and that their exposure can be at or above the safe level (TDI), indicating a potential health concern.

The current study focusses on oats and oat food products and assesses the bioaccessibility and risk of exposure to T-2, HT-2 and HT-2-Glc as the most important oat-derived mycotoxins. The samples analysed are limited to a narrow geographical range (Scottish oats, porridge samples from local supermarkets) and focussed on oat food categories that match the categories at risk of exceeding the recent EU maximum permitted levels for T-2/HT-2 [16].

Future studies are needed to address mycotoxin contamination in oats from a larger geographical area and investigate the effect of different farming practices on fungal infection and mycotoxin contamination. Future work needs to further assess the bioaccessibility of a wider range of regulated mycotoxins and from a wider range of food products and assess the effect of food preparation methods and dietary fibre content on bioaccessibility of mycotoxins. Exposure and co-exposure to multiple mycotoxins need to be assessed in a wider range of foods co-contaminated with other regulated mycotoxins such as DON and ZEN.

## 3. Conclusions

In summary, the oat supply chain faces a real problem with mycotoxin contamination and especially T-2/HT-2 toxins. During processing, de-hulling is a highly effective mitigation strategy to lower mycotoxin contamination by >90%, but residual mycotoxin levels can still lead to some final food products being contaminated with these mycotoxins. Given the high prevalence of T-2/HT-2 in oats and their high bioaccessibility, repeated exposure is likely to occur in oat food consumers at levels around the TDI, with occasional exceedances of safe levels, especially in children. Further efforts need to focus on understanding and limiting fungal infection and mycotoxin contamination with pre-harvest mitigation strategies being paramount to minimising contamination, avoiding carry-over into foods and hence protecting consumer safety.

## 4. Materials and Methods

### 4.1. Mycotoxins Standards

T-2-toxin (T-2, purity 99.1 ± 1.0%), HT-2-toxin (HT-2, purity 97.3 ± 1.0%), ^13^C_24_ T-2 (purity 98.4 ± 1.0%), ^13^C_22_ HT-2 (purity 96.5 ± 1.0%), diacetoxyscirpenol (DAS, purity 98.0 ± 2.0%), deoxynivalenol (DON, purity 93.4 ± 1.0%), ^13^C_15_ DON (purity 99.0 ± 1.0%, DON-3-β,D-glucoside (DON-Glc, purity 96.0 ± 1.0%), nivalenol (NIV, purity 99.3 ± 1.0%), ^13^C_15_ NIV (purity 98.1 ± 1.0%, zearalenone (ZEN, purity 99.7 ± 1.0%) and ^13^C_18_ ZEN (purity 98.8 ± 1.0%) were purchased from Romer Labs Ltd., Tulln, Austria. DAS-3-α, D-glucoside (DAS-Glc), T-2-3-α,D-glucoside (T-2-Glc) [44] and HT-2-3-β,D glucoside (HT-2-Glc) [45] were obtained from Dr. Mark Busman and Dr. Susan McCormick, Mycotoxin Prevention and Applied Microbiology Unit, USDA-ARS-NCAUR in the USA. NIV-3-β,D-glucoside (NIV-Glc) was obtained from Dr. Tomoya Yoshinari, National Institute of Health Sciences, Japan [46]. ZEN-14-β,D-glucoside (ZEN-Glc) standard used in this study was previously synthesised as part of FSA-funded project FS102101. Purity of synthesized standards was not assessed. Working solutions for all mycotoxins were prepared in acetonitrile (ACN) and stored at 4 °C.

### 4.2. De-Hulling of Unprocessed Food Oats

Unprocessed food oat samples (*n* = 20) were collected as part of a previous farm survey [9] from conventional farms across the cereal-producing areas of Southern, Eastern and Northern Scotland. Oat samples were de-hulled through a lab-scale de-huller (product no #11108, Streckel & Schrader, Hamburg, Germany) to separate hulls and de-hulled kernels (groats) and each fraction milled to a fine powder using a ball mill (Retsch model MM400, Verder Scientific, Hope Valley, UK). Mean fraction weights were recorded as 31.3 ± 4.3% hulls, 59.7± 4.3% groats and 9.1 ± 2.1% waste with incomplete separation.

### 4.3. Retail Survey of Oat Foods

The survey constituted 40 porridge oats (21 samples purchased in May–June 2024 and 19 samples in April–July 2025 to increase sample size) and 5 cereal products for infants (purchased in 2025) from major retailers in the Aberdeen area. UK consumer purchase data (Worldpanel Numerator) were used to select the most-purchased products of three further oat food categories: granola and muesli (*n* = 10), oat biscuits (*n* = 11) and oatcakes (*n* = 11). All oat food samples were stored dry as purchased at room temperature and milled to a fine powder using a ball mill (Retsch model MM400, Vender Scientific, UK).

### 4.4. Mycotoxin Analysis in Unprocessed Oats, Oat Fractions and Oat Food Products

Mycotoxin extraction and LC-MS/MS analysis were performed using an established method with modifications [9]. In brief, 2.0 g milled samples were extracted with 8 mL of extraction solvent (79% acetonitrile, 20% H_2_O, 1% acetic acid) for 90 min, centrifuged and 100 µL of supernatants dried under nitrogen and reconstituted in 200 µL of 10% acetonitrile and 1% acetic acid. Samples were spiked with an internal standard mix (50 ng/mL ^13^C_15_ DON, ^13^C_15_ NIV, ^13^C_24_ T-2, ^13^C_22_ HT-2 and 25 ng/mL ^13^C_18_ ZEN) and 15 µL of extract was injected into a Shimadzu Nexera X2 LC system coupled to a Shimadzu 8060 mass spectrometer with an ESI source. Chromatographic separation was achieved on a Phenomenex Gemini C18 column (150 × 3 mm, 3 µm) using 0.1 mM ammonium acetate (A) and methanol (B). After 2 min at 100% A, solvent B was increased to 100% over 12 min, held for 3 min, and after 15 min there was a 0.1 min step where B was dropped to 0% then held to a total time of 17 min. The flow rate was 400 µL/min with a 15 µL injection. The MS operated in positive and negative ion modes with the following parameters: nebulising gas flow 2.5 L/min, heating gas flow 15 L/min, interface temperature 300 °C, DL temperature 250 °C, heat block temperature 300 °C and drying gas flow 5 L/min. Quantification was performed using multiple reaction monitoring (MRM), against 8-point calibration curves (DON 0.625–500 ng/mL; HT-2 0.3125–250 ng/mL DON-Glc, NIV, NIV-Glc, T-2, T-2-Glc, HT-2-Glc, ZEN, ZEN-Glc 0.1563–125 ng/mL). Matrix-matched calibrations were prepared in the extract of a blank unprocessed oat sample. Eight-point matrix-matched calibration curves (see levels above) were prepared and limit of quantification (LOQ) determined as a signal-to-noise ratio of 10. Two blank oat samples and two spiked oat samples were included in each batch of samples analysed.

Recovery was assessed in triplicate by spiking blank matrix samples (unprocessed oats, porridge oats, granola, biscuit and oatcake, 0.5 g each) with a mycotoxin mix (15 µL in acetonitrile) equivalent to 300.0 ng/g DON, 150.0 ng/g HT-2 and 75.0 ng/g DON-Glc, NIV, NIV-Glc, T-2, T-2-Glc, HT-2-Glc, DAS, DAS-Glc, ZEN, ZEN-Glc. Absolute recovery (%) was calculated for each mycotoxin and matrix, and all results corrected for recovery if acceptable (60–140%). Mycotoxins with recovery outside the acceptable range are reported as presence/absence only.

### 4.5. Bioaccessibility of Mycotoxins from Oat Foods

Two porridge oat samples contaminated with T-2, HT-2 and HT-2 Glc (Table 5) close to the recent maximum permitted levels for T-2+HT-2 in oat foods [17] were selected from a sample archive (retail samples from 2014 and 2015). Oat food samples were stored dry at room temperature, milled to a fine powder and mycotoxins extracted and analysed as described in Section 4.4. Mycotoxin stability was ascertained by repeat analysis of both porridge oat samples resulting in 91.9 ± 12.7% of T-2 and 82.9 ± 9.0% of HT-2 compared to levels measured in 2017 (HT-2-Glc was not assessed in 2017).

#### 4.5.1. Upper GI Digestion Using Infogest 2.0

The milled powder was boiled in water to produce porridge as eaten and then digested through salivary, gastric and duodenal phases according to the Infogest 2.0 protocol [47] with minor modifications. In brief, 2.4 g of each oat porridge was boiled with 12 mL water (40 s, microwave model NNK115WB, 1000 Watts, Panasonic, Bracknell, UK) before adding 14.4 mL of Simulated Saliva Fluid (incubated for 2 min at 37 °C in a shaking incubator, model S150, Stuart Scientific, Stone, UK), followed by 28.8 mL of Simulated Gastric Fluid (pH adjusted to 3, incubated for 2 h) and final addition of 57.6 mL of Simulated Intestinal Fluid (omitting bile acids, pH adjusted to 7, incubated for 2 h). Each oat food sample was digested in triplicate, centrifuged (speed 4000× *g*, 5 min and again at 14,000× *g*, 5 min) and pellets and supernatants were stored at −20 °C. Digestion supernatants (6 mL aliquots) were diluted with 24 mL water, adjusted to pH 6.8 with 1 mM NaOH and cleaned through immunoaffinity columns (VICAM-Waters T-2/HT-2 columns, Biocheck Ltd., St. Asaph, UK). Samples were eluted into 1.5 mL acetonitrile, evaporated to dryness and reconstituted in 200 µL 10% acetonitrile and 1% acetic acid prior to LC-MS/MS analysis.

#### 4.5.2. Lower GI Digestion Using Faecal Batch Cultures

Faecal batch experiments were performed as reported before [36,37] with minor modifications. Each digestion pellet (containing the residual oat food) was suspended in 12 mL anaerobic microbial incubation medium M2 [48] under anaerobic conditions. Each digest suspension was then mixed with 12 mL anaerobic faecal slurry (prepared from 2.4 g fresh faeces suspended in 12 mL anaerobic M2) and incubated in anaerobic, sealed Hungate tubes for 48 h. Tubes were then centrifuged (2000× *g*, 10 min) and supernatants frozen. Defrosted supernatants (6 mL aliquots) were centrifuged (13,000× *g*), diluted with 24 mL water, pH adjusted to 6.8 and cleaned through immunoaffinity columns (VICAM-Waters T-2/HT-2 columns, Biocheck Ltd., St. Asaph, UK). Samples were eluted into 1.5 mL acetonitrile, evaporated to dryness and reconstituted in 200 µL of 10% acetonitrile and 1% acetic acid prior to LC-MS/MS analysis (see Section 4.5.3.). Each oat food sample was digested in triplicate and each digestion pellet incubated with 3 different donor faecal samples separately in duplicate, resulting in 18 faecal batch cultures for each digested oat food.

#### 4.5.3. LC-MS/MS Analysis of Upper and Lower GI Digesta

Reconstituted eluents were analysed for mycotoxins T-2, HT-2, T-2-Glc and HT-2-Glc and their internal standards (^13^C_24_ T-2, ^13^C_22_ HT-2) on a Shimadzu Nexera X2 LC system coupled to a Shimadzu 8060 mass spectrometer fitted with an electrospray ionisation (ESI) source (Shimadzu, Kyoto, Japan). A Zorbax Eclipse XDB-C18 (4.6 mm × 150 mm 5-Micron) column with guard column was used for chromatographic separation. Solvents were 0.1 mM ammonium acetate (Solvent A) and methanol (Solvent B). The gradient started at 30% B, increasing to 100% B in 12 min. This was held for 3 min, decreased to 30% B in 0.1 min then held to a total run time of 17 min. The flow rate was 400 µL/min and injection volume was 15 µL. Positive ion mode was used for the mycotoxins of interest with the following parameters: nebulising gas flow 2.5 L/min, heating gas flow 15 L/min, interface temperature 300 °C, DL temperature 250 °C, heat block temperature 300 °C and drying gas flow 5 L/min. Mycotoxins were quantified using multiple reaction monitoring (MRM) set against 8-point calibration curves. Blank matrix samples were produced from a blank oat porridge sample digested via upper GI and lower GI incubations, cleaned through immunoaffinity columns and reconstituted as described in Section 4.5.1 and Section 4.5.2. Limit of quantification (LOQ) was determined in 5-point matrix-matched calibration curves (T-2 and T-2-Glc 0.31–62.5 ng/mL, HT-2 and HT-2-Glc 0.63–125.0 ng/mL) as signal-to-noise ratio of 10. LOQ was determined as 1.25 ng/mL for T-2 and T-2-Glc (upper and lower GI digesta) and 2.5 ng/mL for HT-2 and HT-2-Glc (upper and lower GI digesta).

Digested oat porridge was spiked at levels equivalent to 33.75 ng/g porridge for T-2 and T-2-Glc and 67.5 ng/g porridge for HT-2 and HT-2 Glc to determine recovery. All results for upper GI digestions were corrected for recovery (in spiked oat food digesta: 81.9 ± 0.1% for T-2, 79.6 ± 1.2% for HT-2, 63.3 ± 0.1% for T-2-Glc, 65.0 ± 0.7% for HT-2-Glc). Digestion pellets were spiked at levels equivalent to 33.75 ng/g porridge for T-2 and T-2-Glc and 67.5 ng/g porridge for HT-2 and HT-2 Glc to determine recovery. All results for lower GI digesta were corrected for recovery (in spiked digestion pellets: 66.0 ± 4.6% for T-2, 74.5 ± 7.5% for HT-2, 53.4 ± 5.4% for T-2-Glc, 56.0 ± 5.4% for HT-2-Glc).

#### 4.5.4. Calculations of Bioaccessibility

Mycotoxin bioaccessibility (%) was calculated using the following equation: (total amount of mycotoxin in supernatant obtained in the end of intestinal phase/total amount of mycotoxin in each food sample) × 100, taking into account the different dilution processes during sampling preparation.

#### 4.5.5. Simplified Exposure Assessment

Mean concentrations for T-2, HT-2 and HT-2-Glc in 40 porridge samples (lower bound, values <LOQ replaced by 0, Table 3) were converted to T-2 equivalents and hypothetical exposure calculated either as sum of free T-2 equivalents (T-2+HT-2) or as sum of free + glucosylated T-2 equivalents (T-2+HT-2+HT-2-Glc). Portion sizes were set at 20 g for children and 40 g for adults and body weight set at 18 kg for children [42] and 70 kg for adults. Probable daily intake (PDI) of T-2 equivalents was estimated for each scenario and hazard quotients (HQ) calculated by comparing the PDI (µg/kg bw/d) to the group-based tolerable daily intake (TDI) of 0.02 µg/kg bw/d [41].

### 4.6. Statistical Analysis

Data analysis was performed in GraphPad Prism 11.0.1. Data on mycotoxin levels in unprocessed oats, oat hulls, oat groats as well as porridge oats, granola and muesli, oat biscuits, oatcakes and infant cereal products are presented in tables as mean of all samples with values <LOQ replaced with 0 (lower bound estimates). Mean mycotoxin levels in porridge oats purchased in 2024 and 2025 and were compared using Student’s *t*-test.

## Figures and Tables

**Table 1:** EC maximum levels of selected mycotoxins applicable to oats and oat products.

Mycotoxin	Oat Product	Maximum Level (µg/kg)
T-2+HT-2 ^1,2^	Unprocessed oat grains	1250
	Oats placed on the market for the final consumer; milling products of oats; oat flakes; bakery wares containing at least 75% milling products of oats	100
	Bakery wares except products listed above (containing less than 75% milling products of oats)	20
	Breakfast cereals containing at least 40% milling products of oats and whole grain oats	75
	Breakfast cereals containing less than 40% milling products of oats and whole grain oats	50
	Cereal-based foods for infants and young children	10
DON ^1,3^	Unprocessed oat grains	1750
	Cereals placed on the market for the final consumer	750
	Bakery wares, cereal snacks, breakfast cereals	400
	Cereal-based foods for infants and young children	150
ZEN ^1^	Unprocessed cereal grains	100
	Cereals, bran, cereal flour placed on the market for the final consumer	75
	Bread, pastries, biscuits, cereal snacks, breakfast cereals	50
	Cereal-based foods for infants and young children	20

^1^ EC Reg 2023/915 [16]; ^2^ Amended by EC Reg 2024/1038 [17]; ^3^ Amended by EC Reg 2024/1022 [18].

**Table 2:** Mycotoxins in unprocessed whole grain oats, oat hulls and oat groats.

	Prevalence (% >LOQ)			Mean Concentration (min, max) as ng/g Sample ^a^			Reduction Factor ^b^	
Mycotoxin	Whole Oats	Hull	Groat	Whole Oats	Hull	Groat		LOQ
**T-2**	95.0	90.0	55.0	100.2 (8.2, 633.2)	202.4 (8.0, 819.1)	4.6 (5.2, 24.7)	93.4 (76.4, 100.0)	3.1
**HT-2**	95.0	100.0	55.0	345.7 (33.2, 1503.0)	866.7 (10.4, 3133.9)	7.2 (7.6, 31.2)	98.2 (93.6, 100.0)	6.3
**HT-2-Glc**	90.0	80.0	45.0	244.1 (15.2, 774.5)	410.6 (70.6, 1156.3)	9.4 (13.6, 28.9)	95.5 (80.1, 100.0)	12.5
**T-2-Glc**	85.0	90.0	25.0	137.6 (17.0, 497.2)	330.8 (20.7, 1177.9)	3.7 (12.6, 17.9)	97.3 (83.9, 100.0)	12.5
**DON**	70.0	65.0	10.0	324.9 (36.5, 2325.4)	535.2 (56.9, 2407.9)	37.5 (260.5, 452.5)	92.8 (29.3, 100.0)	12.5
**DON-Glc**	45.0	35.0	20.0	51.0 (15.0, 832.0)	45.0 (12.6, 601.7)	13.6 (18.2, 191.9)	77.1 (47.6, 100.0)	12.5
**NIV**	40.0	75.0	15.0	46.0 (47.1, 327.8)	129.3 (50.3, 619.3)	8.0 (48.5, 51.8)	89.2 (44.2, 100.0)	12.5
**NIV-Glc**	15.0	20.0	0.0	7.0 (25.2, 72.6)	14.6 (40.3, 112.5)	ND	100.0 (100.0, 100.0)	12.5
**ZEN**	15.0	25.0	5.0	4.3 (14.9, 54.0)	9.2 (8.1, 59.9)	0.4 (7.8, 7.8)	91.9 (67.5, 100.0)	6.3
**ZEN-Glc**	0.0	0.0	0.0	ND	ND	ND	ND	6.3

^a^ Concentrations presented as mean of all samples, values <LOQ replaced with 0 (lower bound, LB). Range (min, max) given for values >LOQ. ^b^ Reduction factors calculated as % mycotoxin removed in hull. Reduction factors for NIV-Glc are exclusively based on detection in hull as all groat samples were <LOQ.

**Table 3:** Mycotoxin contamination of porridge oat samples (*n* = 40).

	Prevalence in Porridge Oat Samples	Concentration Porridge Oat Samples
Mycotoxin	% >LOQ (*n* = 40)	ng/g (Min, Max) ^a^
**T-2**	90.0	9.4 (3.2, 29.2)
**HT-2**	67.5	16.9 (7.9, 78.0)
**T-2+HT-2**	90.0	26.3 (3.5, 91.2)
**T-2-Glc**	0.0	ND
**HT-2-Glc**	35.0	9.7 (12.7, 98.9)
**DON**	12.5	18.5 (15.4, 283.9)
**NIV**	10.0	2.3 (17.8, 36.8)
**ZEN**	0.0	ND
**ZEN-Glc**	2.5	0.7 (28.5, 28.5)

^a^ Concentrations presented as mean of all porridge oat samples as purchased, values <LOQ replaced with 0 (lower bound, LB). Range (min, max) given for values >LOQ.

**Table 4:** Mycotoxin contamination of oat food samples.

	Granola and Muesli		Oat Biscuits		Oatcakes		Infant Cereals	
Mycotoxin	Prevalence % (*n* = 10)	Mean ^a^ ng/g (Min, Max)	Prevalence % (*n* = 11)	Mean ^a^ ng/g (Min, Max)	Prevalence % (*n* = 11)	Mean ^a^ ng/g (Min, Max)	Prevalence % (*n* = 5)	Mean ^a^ ng/g (Min, Max)
**T-2**	40.0	3.5 (7.1, 12.6)	27.3	1.7 (5.6, 6.4)	45.5	5.0 (3.3, 22.8)	0.0	ND
**HT-2**	40.0	6.1 (11.7, 20.2)	36.4	4.5 (10.1, 16.7)	63.6	12.5 (6.6, 46.0)	0.0	ND
**T-2+HT-2**	40.0	9.6 (18.8, 32.9)	36.4	6.2 (10.8, 22.3)	72.7	17.4 (6.4, 68.8)	0.0	ND
**T-2-Glc**	0.0	ND	0.0	ND	0.0	ND	0.0	ND
**HT-2-Glc**	40.0	15.9 (24.8, 60.5)	37.3	4.5 (11.4, 20.6)	27.3	11.2 (29.4, 63.4)	0.0	ND
**DON**	0.0	ND	0.0	ND	0.0	ND	0.0	ND
**NIV**	0.0	ND	0.0	ND	0.0	ND	0.0	ND
**ZEN**	0.0	ND	0.0	ND	0.0	ND	0.0	ND
**ZEN-Glc**	0.0	ND	0.0	ND	0.0	ND	0.0	ND

^a^ Concentrations presented as mean of all samples, values <LOQ replaced with 0 (lower bound, LB). Range (min, max) given for values >LOQ. DAS, DAS-Glc, DON-Glc and NIV-Glc were monitored but not quantified (recovery was outside acceptable levels). NIV-Glc was detectable in one sample; DAS, DAS-Glc and DON-Glc were not detectable in any sample.

**Table 5:** Levels of T-2, HT-2 and HT-2-Glc in two porridge oat samples and their bioaccessibility upon in vitro digestion in the upper and lower GI compartments.

		Mean Concentration (ng/g ± SD)	Bioaccessibility (%)	
	Mycotoxin	Undigested Porridge	Upper GI	Lower GI
Oat porridge 1	**T-2**	39.0 ± 2.7	81.9 ± 3.9	3.4 ± 0.3
	**HT-2**	83.2 ± 9.0	64.5 ± 3.0	6.6 ± 1.1
	**HT-2-Glc**	67.0 ± 8.7	96.2 ± 11.8	ND
	**Sum T-2 equivalents**	186.8 ± 15.7	77.4 ± 2.1	4.4 ± 1.2
Oat porridge 2	**T-2**	33.7 ± 1.2	75.4 ± 7.4	2.7 ± 0.2
	**HT-2**	52.9 ± 3.2	57.6 ± 4.4	7.2 ± 1.1
	**HT-2-Glc**	26.6 ± 0.7	89.3 ± 11.4	ND
	**Sum T-2 equivalents**	115.5 ± 5.1	68.9 ± 3.0	4.5 ± 1.2

Data presented as mean ± SD of triplicate extractions for each porridge oat sample. Bioaccessibility calculated as % of undigested porridge and presented as mean ± SD from triplicate in vitro digestions for the upper and lower GI compartments for each individual mycotoxin and for the sum of T-2 equivalents.

**Table 6:** Simplified dietary exposure assessment to T-2 equivalents in children and adults.

	Hypothetical Exposure Children	Hypothetical Exposure Adults
Portion size (g/d)	20	40
TDI (ng/kg bw/d)	20	20
PDI free T-2 equivalents (ng/kg bw/d)	30.9	16.0
PDI free + glucosylated T-2 equivalents (ng/kg bw/d)	39.0	20.2
HQ free T-2 equivalents	1.5	0.8
HQ free + glucosylated T-2 equivalents	2.0	1.0

TDI = tolerable daily intake, PDI = probable daily intake, HQ = hazard quotient, calculated as TDI/PDI. Free T-2 equivalents were calculated as T-2+HT-2, free + glucosylated T-2 equivalents were calculated as T-2+HT-2+HT-2-Glc (as no T-2-Glc was detected in any oat porridge sample).

## Data Availability

The data presented in this study are available on request from the corresponding author. The data are not publicly available due to industry collaboration.
